# Tissue Engineering the Cornea: The Evolution of RAFT 

**DOI:** 10.3390/jfb6010050

**Published:** 2015-01-22

**Authors:** Hannah J. Levis, Alvena K. Kureshi, Isobel Massie, Louise Morgan, Amanda J. Vernon, Julie T. Daniels

**Affiliations:** Department of Ocular Biology and Therapeutics, UCL Institute of Ophthalmology, 11-43 Bath Street, London, EC1V 9EL, UK; E-Mails: a.kureshi@ucl.ac.uk (A.K.K.); i.massie@ucl.ac.uk (I.M.); louise.morgan@ucl.ac.uk (L.M.); a.vernon@ucl.ac.uk (A.J.V.); j.daniels@ucl.ac.uk (J.T.D.)

**Keywords:** cornea, epithelium, endothelium, tissue engineering, repair, RAFT, collagen, GMP

## Abstract

Corneal blindness affects over 10 million people worldwide and current treatment strategies often involve replacement of the defective layer with healthy tissue. Due to a worldwide donor cornea shortage and the absence of suitable biological scaffolds, recent research has focused on the development of tissue engineering techniques to create alternative therapies. This review will detail how we have refined the simple engineering technique of plastic compression of collagen to a process we now call Real Architecture for 3D Tissues (RAFT). The RAFT production process has been standardised, and steps have been taken to consider Good Manufacturing Practice compliance. The evolution of this process has allowed us to create biomimetic epithelial and endothelial tissue equivalents suitable for transplantation and ideal for studying cell-cell interactions *in vitro*.

## 1. The Cornea and Limbus 

At the front of the eye the crystal-clear cornea is exquisitely designed to combine two important functions. The cornea provides the majority of the eye’s focusing power and it also protects the eye, resisting physical insults and preventing entrance of debris and infection. To perform these roles the cornea combines durability and strength with glass-like transparency and smoothness. To maintain clarity the cornea must resist inflammation, vascularization, and invasion by surrounding cells and this requires continuous active participation from the different parts forming this deceptively simple structure. 

**Figure 1:**
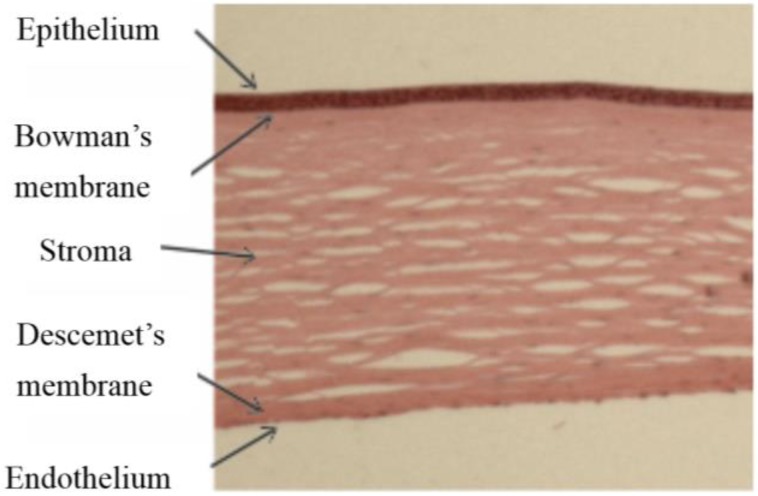
Haematoxylin and eosin stained section of human cornea comprising five layers.

The cornea comprises five layers; stratified epithelium covers the anterior corneal surface facing the external environment, it provides a barrier to infection and permits the flow of fluids and nutrients from the tear film to the main body of the cornea, the stroma or substantia propria. Between the stroma and the epithelium is the Bowman’s membrane, a layer of extracellular protein containing the sub-basal nerves. The stroma itself is a highly ordered collagen structure, produced by keratocytes, the resident cells of the stroma and transparency depends on keratocytes remaining quiescent. At its posterior face the cornea is lined with a specialised endothelium which maintains the stromal hydration levels and thus clarity by pumping fluid out of the stroma into the anterior chamber when required. The endothelium sits on the Descemet’s membrane (DM), another extracellular matrix layer lying between the stroma and the endothelium, it is secreted by the endothelium and essential for endothelial integrity (Figure 1).

At the corneal periphery where the stroma attaches to the tough, white fibrous membranes of the sclera and the clear corneal epithelium gives way to the opaque mucous-secreting conjunctival epithelium, lies the corneoscleral junction or limbus, a unique region with remarkable properties. The limbus provides a rich environment where the stem cells necessary for normal turnover of the corneal epithelium and their support cells reside [1]. In addition, stem cells that are the precursor cells for stromal keratocytes are found near the limbus [2]. The limbus is richly vascularised and contains many myelinated nerves, interestingly the limbus appears to create a barrier, as on its corneal side there are no blood vessels or myelinated nerves. The limbal epithelial stem cells (LESCs) reside in a specialised 3D microenvironment, physically protected from surface insult by lying deep within structures termed limbal crypts (LCs) [3], which are the valleys formed between stromal ridges. The LCs are predominantly located at the superior and inferior regions of the limbus where cells are afforded additional protection from the eyelids [4]. 

## 2. Limbal Stem Cell Deficiency 

Minor surface injuries to the cornea generally heal well in the healthy eye. In cases where the LESCs are compromised or lost, however, conjunctival epithelial growth extends beyond the limbal barrier, replacing the corneal epithelium with an opaque layer that is irregular and heavily vascularised, a painful condition that leads to sight loss. Limbal epithelial stem cell deficiency (LESCD) can be the result of damage to the eye following physical trauma, burn or chemical insult, a genetic disorder, as seen in aniridia generally caused by loss of function of the PAX6 gene [5] or other diseases such as the autoimmune Stevens-Johnson syndrome [1,6]. 

## 3. Current Treatments for Limbal Stem Cell Deficiency 

Grafts of LESCs can be applied to the corneal surface to treat patients with LESCD. To prepare the cornea before placing the graft and to improve the chances of graft survival the surface of the cornea must be repaired by removal of the conjunctiva from the corneal wound bed and inflammation controlled before delivering the LESCs. Donor stem cells are then attached to the front of the eye and cells can be applied directly as tissue grafts or after *in vitro* expansion. Cultured LESCs can be applied simply as a sheet of cells, or prepared on a scaffold that is grafted on to the ocular surface with the cells attached. Human amniotic membrane (HAM) and fibrin are both commonly used as scaffolds to deliver LESCs to the corneal surface in the clinic [7,8]. HAM is prepared from donated tissue after elective caesarean section. It has been widely used as a carrier for limbal stem cell grafts as it provides an efficient scaffold for cell seeding and has other beneficial biological properties including anti-inflammatory, anti-microbial, anti-fibrotic, anti-scarring, and low immunogenicity, as well as good mechanical properties.

## 4. Drawbacks to Current Treatment Strategies 

HAM is highly biologically variable [9] and often grafts destined for patient transplantation have to be discarded due to lack of limbal epithelial stem cell growth on the surface. HAM also has the potential to carry infection, and so must undergo screening before use, and, thus, is sub-optimal for cell therapy Good Manufacturing Practice (GMP) compliance. A biomimetic replacement for HAM would address an unmet clinical need and improve availability of treatment, as supplies of suitably processed HAM are limited. Fibrin gels provide a good alternative substrate for limbal epithelial stem cell growth and can be used as a carrier for graft cells. However, their degradation rate must be tightly controlled with the addition of anti-fibrinolytics [10].

## 5. Requirements for a Successful Cell Carrier

The essential properties of a cell carrier include optimal human limbal epithelial (hLE) cell culture and attachment to the surface, it must be capable of being attached to the cornea by sutures or fibrin glue and once on the eye it should either be resorbed, leaving cells well attached (fibrin) or become transparent *in situ* (HAM). Most importantly, it is essential that a proposed carrier does not initiate an inflammatory reaction in the corneal surface that might trigger scarring and haze in the stroma. Where the eye surface is damaged, the use of a scaffold like HAM with considerable mechanical strength can help to stabilise the ocular surface, as well as acting as a delivery vehicle for therapeutic stem cells. Graft cells can be of limbal origin and either autologous or allogeneic. Autologous is preferable and possible if one healthy eye remains but allogeneic cells are required from a suitable donor if the damage is bilateral leaving no healthy limbus for autograft. In addition, successful grafts have been made from other stem cell sources, notably from oral mucosal epithelium [11]. 

## 6. Alternative Cell Carriers for Transplantation 

In addition to HAM and fibrin, many biomaterials and synthetic polymers have been developed with a view to making tissue equivalent scaffolds for delivering stem cells and for surface repair in the cornea. These include keratin, silk fibroin, chitosan hydrogels [12], siloxanehydrogel contact lenses, thermo reversible polymers [13] and nanofibre scaffolds, recently reviewed by He and Yiu [14] and Feng *et al.* [15]. In addition to these alternatives, extracellular matrix (ECM) based scaffolds can act as suitable substrates for tissue engineering due to their inherent properties of biocompatibility and degradability. Collagen is the most abundant ECM protein in the human body, providing structural support and strength to various tissues. Specifically, type I collagen is the most abundant collagen of the cornea, and its highly organised collagen ultrastructure is crucial in providing structural support and transparency to the tissue. Type I collagen has widespread use in tissue engineering applications due to its abundance, ease of extraction and adaptability for multiple applications [16]. We describe here our efforts over the preceding six years to develop a novel collagen-based tissue engineering approach that is applicable to the treatment of corneal defects in both the epithelium and endothelium. 

**Table 1:** Summary of advantages and disadvantages of various alternative scaffolds for transplantation.

Scaffolds	Advantages	Disadvantages
Keratin	good transparency; good mechanical strength; good availability	limited elasticity; no *in vivo* data; no data regarding clinical efficacy; cannot incorporate cells within
Silk fibroin	non-immunogenic; degrades *in vivo*; biocompatible; good transparency; good mechanical strength; well characterised (already used as a suture material); good surface for hLE cell expansion *in vitro*	costly to produce; no data regarding clinical efficacy; cannot incorporate cells within
Siloxane hydrogel	good mechanical properties good transparency well characterised (already used as contact lenses) good surface for hLE cell expansion *in vitro* clinical data is encouraging	cannot incorporate cells within
Fibrin	good mechanical properties; degrades *in vivo*; good surface for hLE cell expansion *in vitro*; clinical data is encouraging; could potentially be an autologous therapy	reported transparencies vary; risk of disease transmission; cannot incorporate cells within; some evidence to suggest that hLE differentiation is promoted on fibrin
Thermo reversible polymers	good surface for hLE cell expansion *in vitro*; simple process to harvest hLE cell sheet	hLE must be transplanted as a sheet, surgically complex; cannot incorporate cells within
Nanofibre scaffolds	good mechanical properties; good transparency; good surface for hLE cell expansion *in vitro*; may degrade *in vivo*	no data regarding clinical efficacy; mechanical properties may change throughout hLE culture period; cannot incorporate cells within
Chitosan hydrogels	well characterised (already used as wound dressing); good mechanical properties	limited transparency; cannot incorporate cells within; methods used to improve mechanical properties may increase cytotoxicity

## 7. Plastic Compressed Collagen 

**Figure 2:**
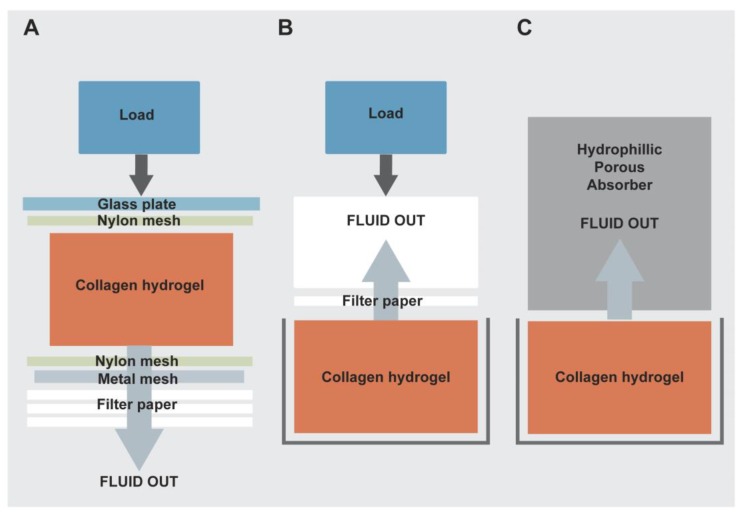
Evolution of the RAFT tissue engineering procedure. (**A**) Schematic diagram of original plastic compression process with application of a load for unconfined compression and downward fluid flow; (**B**) Confined compression in a well plate with upward flow on application of a load; (**C**) Current RAFT process with gentle wicking of fluid into HPAs in a confined manner with no addition of a significant load.

Cellular type I collagen hydrogels have previously been used as 3D substrates for cell culture and creation of corneal models. However, because they are composed of a high proportion of water they are intrinsically weak unless modified with chemical crosslinking or blended with other polymers to create collagen composites, preventing direct seeding of cells within the scaffold [17,18,19]. To overcome this problem, Brown and colleagues developed a novel method of plastic compression of type I collagen hydrogels by applying simple engineering techniques such as external mechanical loading and capillary fluid flow (Patent number WO2012004564) [20,21]. Hyperhydrated collagen gels are composed of a mesh of collagen fibrils supporting a large volume of excess fluid (99%). Hydrogels were produced by neutralising a mix of acetic acid based rat-tail type I collagen and 10× minimum essential medium (included as an indicator of pH and to ensure physiological ionic strength for cell compatibility and standardised collagen fibril formation) with sodium hydroxide. Liquid was then cast into moulds and set/stabilized at 37 °C. In their method, hydrogels were subjected to an unconfined compression by placing the gel on top of a series of nylon and stainless steel meshes and blotting filter papers with addition of a load (Figure 2A). The key feature of this process is that the expulsion of this liquid does not return on the removal of the load, hence the hydrogel undergoes plastic compression (PC) in an unconfined manner. One major advantage of this method is that cells can be seeded into the body of the hydrogel before compression and therefore production of a cell seeded scaffold is ultrarapid, taking just minutes and without loss of cell viability. The use of plastic compressed collagen as a substrate/scaffold has been proposed for many tissue engineering applications, including bone [22], skin [23], nerve [24], bladder [25], and microvascular endothelium [26]. 

We exploited the Brown method to produce a novel substrate for hLE cell expansion and stratification into a corneal epithelial tissue equivalent (TE) [27]. Collagen type I hydrogels were subjected to a process of PC as seen in Figure 2A. In order to determine the importance to the epithelial layer of closely associated stromal cells, we compared acellular constructs with those containing human limbal fibroblasts (hLFs) in the body of the construct, mimicking the hLF populated anterior stroma of the cornea. These cells were mixed directly into the collagen hydrogel solution before setting and compression. hLE cells were then seeded onto the surface of the constructs and allowed to expand and stratify over a period of three weeks. The resulting corneal epithelial TE’s displayed many of the characteristics of human corneal epithelium. These included typical morphological organisation of cuboidal basal cells with high levels of putative stem cell marker (p63α) expression, squamous superficial cells with high levels of CK3 expression and appropriate basement membrane protein deposition. The hLF containing constructs displayed increased biomimicity suggesting an interaction between hLF and hLE cells. The resulting TEs provide an ideal model for studying cell-cell interactions in the anterior cornea and made steps towards development of a suitable alternative to HAM for hLE cell transplantation for ocular surface repair. 

Although this technique fulfilled many of the criteria for a successful alternative substrate for HAM including ease and speed of preparation and relative transparency there was a major drawback associated with this method because of the unconfined nature of the compression process. This meant that fluid loss was not controlled and, thus, the procedure was practically messy and, thus, did not comply with the rigorous GMP standards required for production of a cell therapy. Secondly, in this format the method was difficult to scale up for easy production of multiple constructs, therefore, we went on to modify the process in order to make steps towards GMP compliance and scalability.

## 8. The Early RAFT Process 

**Figure 3:**
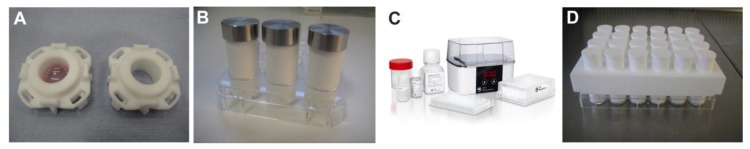
Evolution of the RAFT process hardware. (**A**) Individual cassette for upward confined compression method; (**B**) Paper rolls and weights used for 12 well plate confined compression method using upward flow; (**C**) Commercially available RAFT kits including reagents, plates and plate heater (image reprinted with permission of TAP Biosystems); (**D**) 24-well plate array of HPAs.

In collaboration with a SME (small and medium sized enterprise; TAP Biosystems), we developed what we now refer to as the Real Architecture For 3D Tissues (RAFT) process by extensive modification of the original PC process from a variable hand-made research method [20] to an improved, reproducible process suitable for clinical application [28]. The differences between the three iterations of the process are summarised in Table 2. The original unconfined compression (UC) process was not tightly controlled and therefore not compatible for use in a GMP clean room facility. In particular, this process had a greater potential for production of aerosols and particulates, which is not ideal for a GMP compliant approach. Additionally, the final product had inconsistent dimensions due to fluid loss in various directions. To overcome these issues, the collagen hydrogel mixture was cast, set and compressed in a confined manner using a custom-made cassette or 12-well plate format (Figure 3A,B). The modification involved a change in the direction of fluid removal to one of upward flow using a rolled filter paper absorber and other blotting elements placed on the top surface of collagen hydrogels for 15 min at room temperature (Figure 2B and Table 2). As a weight was applied, hydrogels still underwent compression but this time it was a confined compression (CC), which reduced the risk of aerosol and particulate production. Following compression, RAFT TEs were ready in a multi-well plate format or an individual cassette for immediate analysis or subsequent culture of cells. This reduced the risk of damaging RAFT TEs during handling or transfer from one vessel to another as was required for the original PC method. 

**Table 2:** Summary of the three iterations of our tissue engineering protocol.

Iterations	Fluid removal method	Fluid removal direction	Confined or unconfined	Absorbent material
Plastic compression	Compression with load	Downward	Unconfined	Filter paper
Early RAFT	Compression with load	Upward	Confined	Filter paper
Late RAFT	Absorption	Upward	Confined	Hydrophilic porous absorber

We investigated whether these modifications (from UC to CC) affected the properties of RAFT TEs and their ability to act as a substrate for culture of hLE cells [28]. Comparison of RAFT TEs prepared with either UC or CC revealed no differences in hLE cell morphology or phenotype. hLE cells remained small in size with characteristic cobblestone morphology and expressed putative stem cell marker p63α and differentiated epithelial cell marker CK15. Cells also expressed features similar to mature human corneal epithelium (CK3). There were no significant differences in the light transmission properties, handling and cell viability of RAFT TEs produced using UC and CC processes. The thickness of RAFT TEs was the only parameter that was significantly different, with thicker substrates produced by the UC process. However, a change in this property did not adversely affect the function of RAFT TEs as a substrate for hLE cell culture and was thought to be due to an increased wicking power in the CC process resulting in greater fluid loss and a thinner TE. Importantly, the thickness of RAFT TEs produced with CC was consistent, unlike HAM, which varies greatly ranging from 20–130 μm [29,30,31].

We also described the first use of RAFT TEs, prepared using CC, to successfully support the culture of primary human corneal endothelial cells (hCECs) in addition to corneal endothelial cell lines [32]. Corneal blindness caused by a dysfunctional endothelial layer was previously treated by full thickness corneal replacement surgery but recent advancements in surgical techniques allow the replacement of only the affected endothelial layer. One such example is replacement of a defective endothelium with a Descemet’s Membrane Endothelial Keratoplasty (DMEK) graft, a technique that delivers a healthy endothelial cell layer attached to just the delicate DM layer obtained from a donor cornea [33]. However, there is currently a worldwide donor shortage of clinical quality corneas that greatly increases patient waiting times for such procedures and this has led to considerable interest in the development of alternative tissue engineering approachs to treat such corneal defects [34]. hCECs cultured on RAFT TEs retained their endothelial characteristics, remaining small and with cobblestone morphology and expressed ZO-1 and Na^+^/K^+^ ATPase; markers typically used for the identification of hCECs. They were also easy to handle and demonstrated sufficient mechanical strength for delivery into the anterior chamber of an *ex vivo* porcine eye, indicating suitability for transplantation. This approach has great potential to improve clinical practice as only one human donor cornea is required to obtain a sufficient number of hCECs to treat multiple patients, unlike the full thickness or DMEK grafts described earlier that require one donor per recipient. 

The advantages of the CC RAFT process included simple, rapid production of multiple TEs with limited variation, as well as making considerable progress towards GMP compliance. The use of individual cassettes was ideal for preparation of patient grafts for transplantation because all three elements of manufacture, cell culture, and TE transport to theatre were performed within the same vessel. The use of commercially available multi-well plates to perform the RAFT process made it an attractive approach for pharmacological testing of substances on multiple TEs. It also enabled the process to be scaled up into 24-well and 96-well plate formats, further reducing preparation time without compromising reproducibility.

## 9. Improved Good Manufacturing Practice Compatibility of RAFT

Cell therapy products must be manufactured within a specialised cleanroom laboratory in compliance with GMP [35]. The specific considerations around the design of equipment and processes used in GMP manufacture include the following; the manufacturing process must be reproducible, time-effective, generate a consistent end-product and utilise equipment compatible with cleanroom use and cleaning methods. The process must also include measures to prevent microorganism contamination, such as the use of one-use reagents, smooth-surfaced and low particle shedding materials, and must not present risk to the patient through adventitious agent transmission [35,36]. Therefore, our previous RAFT process was even further modified to its current commercially available form with the above GMP considerations in mind.

The commercially available RAFT kit is provided with validated protocols that are simple to use and a 37 °C plate heater for standardised hydrogel setting and TE formation (Figure 3C). The whole process is robust, reproducible and allows for the manufacture of multiple consistent RAFT TEs. The process is rapid; from initial set-up to the generation of the RAFT TE takes as little as 90 min which is an advantageous attribute for GMP manufacturing. This is because in order to comply with health and safety guidelines, operators are designated a maximum processing time. Additionally, GMP grade manufacturing is inherently costly so any reduction in facility costs is preferable.

The casting of collagen hydrogels in commercially available well plates remains consistent with the previous iteration of the method. However, instead of the CC process, a gentle wicking process is employed whereby water is absorbed through an upward flow capillary action into hydrophilic porous absorbers (HPAs) (Figure 2C, Figure 3D and Table 2). Therefore, the process is no longer one of compression as no significant load is added, only the minimal weight of the HPA itself; fluid is lost through gentle absorption alone. This containment of fluid further minimises contamination risk in keeping with GMP requirements. In addition, the simplification of the procedure provided by the use of disposable HPAs and elimination of re-useable weights, is more appropriate for processing in a GMP environment. 

The HPAs are manufactured from porous sintered polymers and contain a standardised pore size optimised to efficiently absorb liquid. Additionally, HPA polymer materials are non-particle shedding therefore minimising potential contamination risk. The moulding process produces HPAs of a standard size (compatible with 24-well and 96-well plates) and allows for reproducible RAFT TE size every application (Figure 3D). The HPAs are supplied sterile and individually packaged to give ultimate flexibility in producing the number of TEs required without wastage of materials. The RAFT plate heater has been designed with a small footprint to fit through cleanroom hatches and inside biological safety cabinets, and also contains smooth surfaces to allow efficient cleaning. The 96-well plate format, available in both clear and black bottomed varieties, allows for a broad range of applications and the production of multiple RAFT TEs per manufacturing session. The supplied reagents for such an application, e.g., type I collagen, neutralising solution and 10X MEM, are designed for “one-use” only in order to prevent potential contamination risks posed through aliquoting manipulations and storage. 

An important consideration in the development of RAFT reagents was to ensure adherence to EU legislation on the use of animal-derived materials during GMP manufacture [35,36]. Biologically sourced materials may pose increased risk through transfer of adventitious agents such as bacteria, viruses or prions, therefore legislation dictates that such materials must be carefully risk-assessed including how they demonstrate compliance to prescribed controls. RAFT type I collagen has undergone such risk assessments and has been classified “Transmissible Spongiform Encephalopathies (TSE) compliant”, where risk of transmission of TSE-causing agents is negligible due to the sourcing of animals from closed herds and from a country with negligible Bovine Spongiform Encephalopathies (BSE) risk. With respect to concerns of potential viral and bacterial contamination, the herds are screened, certified free from infectious disease and classified fit for human consumption. The collagen reagent itself is provided with quality documentation to demonstrate animal country of origin, TSE compliance and product specification, all of which are necessary documentation for materials involved in GMP manufacture. Each component part of the RAFT process has been assessed and essential GMP criteria have been considered at each developmental stage. 

## 10. Recreating the Limbal Epithelial Stem Cell Niche Using RAFT

**Figure 4:**
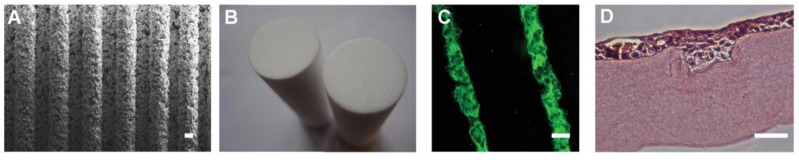
Creation of bioengineered limbal crypts using RAFT. (**A**) SEM image of the base of a RHPA showing microridges; (**B**) Base of HPA (left) and RHPA (right); (**C**) BLCs created in the surface of the RAFT TE filled with fluorescent microspheres; (**D**) Human corneal epithelial cell line cells stained with haematoxylin and eosin filling a BLC in a RAFT TE. Scale bars: (A,C), 100 μm; (D), 50 μm.

We have previously used the RAFT system to create relatively simple central corneal TEs but the flexibility of the HPA system allows us to also create some of the features of the complex hLESC niche. The ability to design and engineer artificial stem cell niches has allowed researchers to simplify elements of complex environments for *in vitro* study and also to maximise the therapeutic potential of stem cells for repair strategies. As previously described, the LESCs reside at the base of the LCs in a physically protected position predominantly at the superior and inferior regions of the limbus [3] and so we hypothesised that culture of cells in a recreation of that physical environment could increase hLESC potency. By studying native LCs in human corneas we were able to determine average dimensions of the LESC filled stromal structures, which are approximately 110 μm wide and 70 μm deep. The moulding method of manufacture of the HPAs enables surface topography to be incorporated into the HPA surface that will contact the collagen gel. We created ridged HPAs (RHPAs) with micro-ridges of varying width and depth (100–250 μm; Figure 4A) to determine the most suitable size to create features in the RAFT TE surface that would mimic the native LCs. We determined that RHPAs with 175 μm micro-ridges would create bioengineered limbal crypts (BLCs) of the desired dimensions and so created HPAs with ridges across the entire surface (Figure 4B). The BLCs remained stable in culture (Figure 4C) and in the presence of hLFs in the stromal portion of the RAFT TE and with hLECs on the surface [37]. Human corneal cell line cells populated the BLCs (Figure 4D) and hLECs also multi-layered to fill the BLCs with cells at the base expressing putative LESC markers as well as appropriate basement membrane proteins. Interestingly, hLFs in the stromal portion of the RAFT TE aligned and elongated along the length of the BLCs (unpublished observations), which mimics the arrangement of stromal cells in this region in close proximity to the native LCs. 

Using the RAFT system we can rapidly and reliably recreate the 3D physical structures of the LESC niche in as little as 90 minutes, an improvement on other lengthier approaches [38,39]. Mimicking the elements of the niche will allow investigators to study cell-cell and cell-matrix interactions in a more biomimetic environment and could potentially increase the chances of success of cell based therapy by maintenance of critical stem cell populations. 

## 11. Clinical Application of RAFT 

The ultimate goal of our research is to treat patients suffering from LESCD. Whilst this has always been a consideration during the development of the RAFT process, in order to be clinically applicable, further considerations must be made. The current “gold standard” substrate for LESC transplantation is HAM [40,41,42], despite the disadvantages outlined earlier. RAFT TEs are reproducible, quick to manufacture, do not require the expensive screening required for human tissue and reliably support hLE cell growth *in vitro* [27,37], all evidence to support the use of RAFT TEs over HAM for ocular surface repair.

During RAFT process development, the initial focus was to optimize hLE cell growth on the surface of the collagen, as opposed to end user requirements such as thickness, transparency and strength. The thickness of the substrate is important so as not to cause discomfort to the recipient or complicate the surgical procedure. Similarly, light transmission is important to provide the recipient with the best possible visual acuity post-transplantation. Although collagenous RAFT TEs may undergo some degree of degradation post-transplantation, at present we have no *in vivo* data to support this, so optimal transparency from the outset is desirable. Finally, RAFT TEs should have sufficient mechanical strength to withstand handling and attachment to an eye using fibrin glue or similar.

One great advantage of the RAFT TE production process is that it is readily tunable. Since the concentration and volume of collagen used to produce the hydrogel can be varied, RAFT TEs with a variety of properties can be produced after fluid absorption using the HPAs. RAFT TEs can be produced which have thicknesses ranging from approximately 50 μm to more than 400 μm. Light transmission at 550 nm (the middle of the visible wavelength range) through RAFT TEs can exceed 80%. Whilst under tension, RAFT TE break stresses are comparable to that of HAM. HAM is already established as a substrate, albeit sub-optimal, for LESC transplantation and we have shown that RAFT TEs of comparable thickness, transparency and strength can be produced, but with additional advantages as outlined above. Additionally, unlike HAM, RAFT TEs display shape memory, which may ease surgical delivery to the ocular surface.

Along with the RAFT TE substrate, the cells themselves should be fit for purpose. For Advanced Therapy Medicinal Products (ATMP), this is normally determined using a reference standard or potency assay. A reference standard provides a comparator with which all manufactured batches can be compared to confirm that the manufacturing process is proceeding as intended/required. For RAFT TEs, we have demonstrated that hLE cell size, morphology, putative stem cell marker expression (p63α) and ability to form a stratified epithelium could be used as part of a reference standard [43]. A correlation between expression of p63α and clinical outcome has also been suggested by others [44], further supporting the use of p63α expression as part of a reference standard. When producing an autologous treatment, this can be complex as only a limited numbers of cells are available but by observing the hLE cells during culture using light microscopy, progress can be assessed using a simple, non-destructive method. Throughout the culture period individual cultures on RAFT TEs may be compared with light microscopy images of the reference standard for assessment of confluency. Beyond this, a potency assay provides a means of testing the function of the ATMP. hLE cells on RAFT should function to protect the underlying stroma by maintaining a continuous barrier. To test hLE cell function, we used two wounding regimes: an Algerbrush II corneal rust ring remover to produce a 1 mm stripe defect [43]; and a heptanol-soaked paper disc to produce a larger 20 mm^2^ circular defect [45]. We found that hLE cells on RAFT were reliably able to close the stripe defect, although p63α expression was decreased following wounding [43]. This is important as this provides evidence that hLE cells are sufficiently potent to be clinically useful. We also assessed the impact of the presence of hLFs on hLE cell function and also the impact of airlifting using the heptanol wounding regime. We found that hLFs do not improve hLE cell potency with respect to wounding (although hLFs do confer other benefits to overlying hLE cell layers as described earlier). However, we did find that hLE cells in a stratified epithelium displayed reduced cell function compared to cells in a monolayer, leading us to believe that transplantation of a monolayer of hLE cells on RAFT TEs would be the optimal approach [45].

## 12. Summary

Corneal disease and disorder is the cause of blindness in over 10 million people worldwide. A lack of suitable donor tissue has lead to increased interest in the development of tissue engineering strategies to construct tissue equivalent layers of the cornea to replace the defective regions. Here we have described the development of our RAFT tissue engineering process that aims to tackle this very problem. Our final RAFT process is very far removed from the original plastic compression process, which required six component parts. After modification of the flow removal method, direction, containment, and absorber material the process now only requires one component, the HPA, to transform a hyperhydrated collagen hydrogel into a mechanically stable substrate for cell growth. This simplification of the method meant that we were able to take considerable steps towards GMP compliance and clinical applicability. We have shown that this method is suitable for the production of both corneal epithelial and endothelial TEs which may be suitable for transplantation. Furthermore, the tunable nature of the HPA production method allowed us to create surface topography in the TEs to rapidly and reliably recreate LESC niche features, the LCs of the cornea. This will allow researchers to study cell-cell and cell-matrix interactions of the LESC niche using a more biomimetic *in vitro* model. We have taken a basic research process and refined it for translational use so that it has the potential to be delivered to patients to treat ocular surface failure within the next two years. 
